# State of Prestressing Analysis of 62-Year-Old Bridge

**DOI:** 10.3390/ma15103583

**Published:** 2022-05-17

**Authors:** Jakub Kralovanec, Frantisek Bahleda, Martin Moravcik

**Affiliations:** 1 Department of Structures and Bridges, Faculty of Civil Engineering, University of Zilina, Univerzitna 8215/1, 010 26 Zilina, Slovakia; martin.moravcik@uniza.sk; 2 Laboratory of Civil Engineering, Faculty of Civil Engineering, University of Zilina, Univerzitna 8215/1, 010 26 Zilina, Slovakia; frantisek.bahleda@uniza.sk

**Keywords:** bridge, prestressing force, assessment, non-destructive testing, saw-cut method, structural response method, Barkhausen noise technique

## Abstract

Ageing infrastructure leads to the need for a proper assessment and final decisions considering its state. In the case of prestressed concrete structures, knowledge of the residual state of prestressing is the crucial factor. Therefore, reliable diagnostic techniques for determining the residual value of the prestressing force are needed. This information is subsequently used in the process of the quantification of the load-carrying capacity and remaining service life of prestressed concrete structures. The presented paper introduces an evaluation of a monolithic 62-year-old prestressed concrete bridge, which was built in 1959. The assessment was carried out as a result of concerns after exposure of the anchorage area of the bridge, which was executed during the construction of the new system of anti-flood barriers in the town of Banska Bystrica in central Slovakia. Therefore, the diagnostic survey and subsequent determination of the residual prestressing force included the application of the saw-cut method, the structural response method, and the Barkhausen noise technique. Finally, the experimental program supported by numerical analysis provided information about the actual state of prestressing in the bridge. Results of performed analysis suggested that the state of prestressing of the bridge in question does not significantly differ from the expected level of prestressing after 62 years of service. Subsequently, obtained conclusions enabled the determination of the load-carrying capacity for future use in the form of a pedestrian bridge.

## 1. Introduction

In the Slovak Republic and other European states, we have witnessed the growing importance of the proper assessment of prestressed concrete bridges and structures. As an example, since 2015 at least five prestressed concrete bridges in Slovakia have failed or have been out of service. In most cases, these structures belong to the first generation of precast prestressed concrete bridges which were built in former Czechoslovakia. As a result of their neglected maintenance which should be provided by the administrators, a noticeable reduction of the state of prestressing can be expected in these types of prestressed concrete bridges [1,2].

Prestressed concrete bridges have successfully been established as one of the pillars of infrastructure in many countries. In the Slovak Republic, the first applications of prestressed concrete (PC) date back to the 1950s and 1960s, so bridges from this construction material are inevitably approaching the limit of their service life [1,2,3]. Nowadays, the task of the civil engineers is to decide whether the state of prestressed concrete bridges from the first generation enables them to be preserved in service with minor actions, or if the administrators should be forced to replace them with the new, more technologically advanced structures [4]. The current alarming situation is not only a result of some conceptual errors made by our predecessors, because insufficient maintenance provided by the current administrators significantly contributed to this issue. In the case of Slovak road infrastructure, more than the third of bridges in service do not meet the required criteria for reliability, and 2.50% are in a very poor or breakdown condition. For these reasons, the research activities of the Department of Structures and Bridges (Faculty of Civil Engineering, University of Zilina) include the activities which are concentrating on the assessment and evaluation of bridges and structures in service [4,5,6,7].

At present, we know various direct and indirect approaches for determining the value of residual prestressing force. Commonly, these methods can be categorized according to their impact on the investigated construction or methodology in which the prestressing force is monitored [8,9,10,11]. The techniques with no or only a negligible impact on the structure’s integrity are usually considered non-destructive or semi-destructive. In contrast, destructive methods unavoidably produce damages to the evaluated prestressed concrete structure which can affect the structural integrity. Direct approaches typically fall into a group of non-destructive methods and enable the determination of a residual prestressing force value directly from the results of the measurement. On the contrary, the application of indirect methods, such as the saw-cut method (SCM) and structural response method (SRM), leads to the calculation of the prestressing force indirectly from the measured values. Measured quantities can be represented by, e.g., deflection, stress (or strain) release, the width of the cracks, and so on [10,11,12]. Ongoing research in the field of non-destructive prestressing force monitoring is focused, for example, on vibration methods, impedance-based methods, elasto-magnetic methods, acoustic elastic methods, and strain-based methods. In [13], the authors describe the pros and cons of several methods for monitoring prestress losses. However, in practice from the abovementioned techniques, only strain-based methods are extensively used in field applications due to a complicated calibration and limited possible application [13]. Moreover, the level of residual prestressing can be also non-destructively evaluated by means of the measurement of vertical deflections or a structural response (crack initiation/re-opening) [14].

The presented analysis of a post-tensioned bridge consists of the application of two indirect methods—the saw-cut method and structural response method. Moreover, the analysis includes the verification of obtained results using the Barkhausen noise technique (MBN).

**Figure 1:**
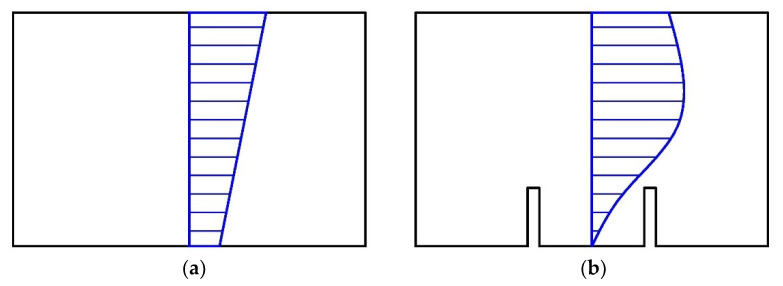
Normal stress distribution in PC member—before sawing (**a**); after sawing (**b**).

The concept of the saw-cut method is the isolation of a concrete block from acting forces caused by saw-cuts. The value of the prestressing force is then derived from the stress (or strain) change monitored in the area adjacent to the performed saw-cuts [15]. If it is possible to determine the percentage of change in the stress or strain, for instance, by employing proven findings of numerical simulations, the full isolation of the concrete block is not strictly required; specifically, in cases of the assessment of ageing PC bridges with a very small value of concrete cover. Consequently, concrete cover limits the maximum depth of the applied intervention [2]. When applying the saw-cut method on an unloaded prestressed concrete structure, the value of the residual prestressing force can be calculated considering the linear relation between normal stress and external forces. Therefore, this approach is applicable only in the case of an uncracked prestressed concrete member with a corresponding linear distribution of stress. Naturally, if the investigated PC structure is loaded also by the external load, additional normal stress resulting from such a load must be taken into account [15]. The principle of the saw-cut method is shown in Figure 1.

**Figure 2:**
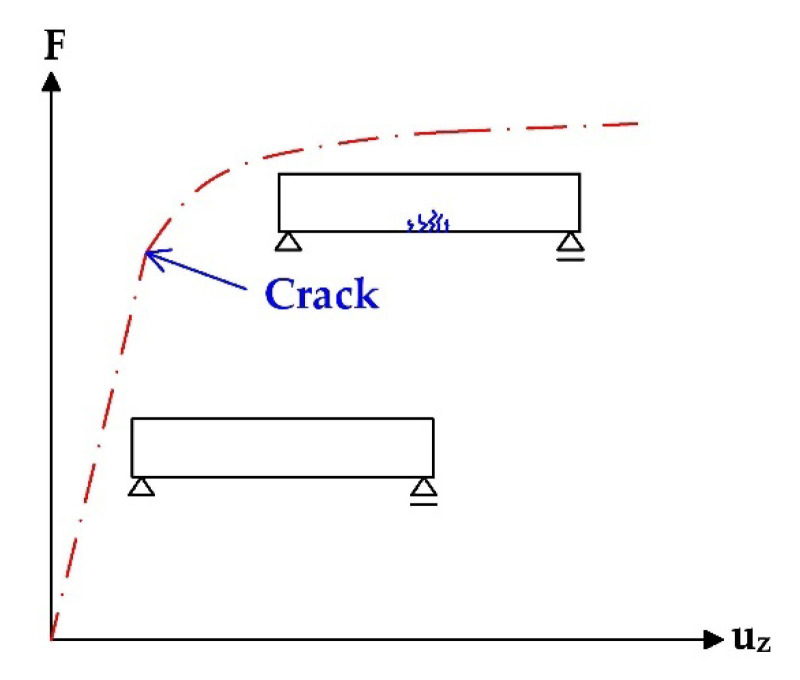
Influence of stiffness decrease on the deflection of PC structure—SRM.

The structural response method is based on the observation of the effect of the load applied on investigated prestressed concrete structures. Usually, we measure deflection and strain. Subsequently, the obtained results are compared to analytically determined values. Thus, the determination of the actual state of prestressing is possible. In the case of the structural response method, the application of the known external load is the most common option (the short-term effect on the structure). However, another possibility is to measure the long-term effect of a dead load, including the rheology of concrete (creep and shrinkage) which is very important in the assessment of prestressed concrete structures. Nevertheless, *time-dependent analysis is a very difficult task when reliable input data are missing.* Consequently, *the determination of residual prestressing in the case of the majority of* existing prestressed concrete *bridges is very difficult due to a lack of* inputs. *Therefore, it is needed to apply assumptions, for example, based on the codes.* An important part of the evaluation of SRM results is the consideration of the beam’s stiffness, as the course of the predicted and actual deflection (or strain/stress) begins to be drastically different after the first crack is formed, see Figure 2 [16].

Magnetic Barkhausen noise (MBN) represents the alternative non-destructive technique in which the stress state in ferromagnetic bodies (such as prestressed wires) can be investigated. The amplitude of MBN tends to increase along with increasing tensile stresses, and decreases with compressive ones when the energy magnetocrystalline anisotropy is more magnetoelastic [17,18]. However, in the case of prestressed wires, when the wires matrix is preferentially oriented along the wire length [18,19,20,21], the energy of magnetocrystalline anisotropy is usually fully consumed and MBN tends to drop down when tensile stress increases [19,21]. It has been already reported that an assessment of the true prestress in the wires can be carried out when the MBN emission in the prestressed wires is measured, followed by the tensile stressing of some wires in the laboratory (after their cutting off). The true value of prestress can be found as the intersection of the calibration evolution and the measured MBN on the bridge. Further details, as well as the methodology, can be found in the previous study [19]. Having experience with MBN emission, this technique was also integrated into our study in order to confirm calculations associated with the wires prestressing.

## 2. Diagnostic Survey

**Figure 3:**
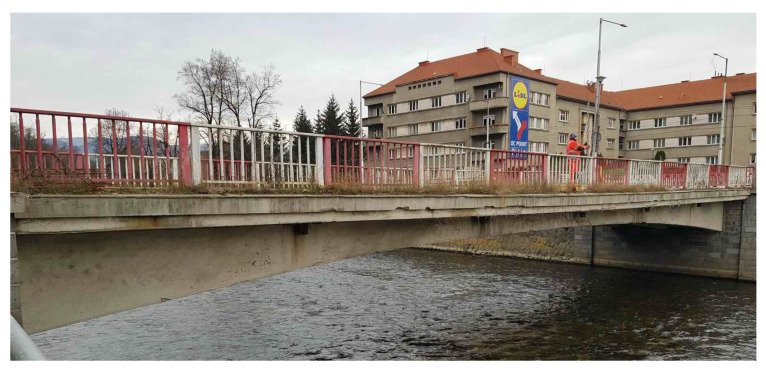
View of the bridge.

**Figure 4:**
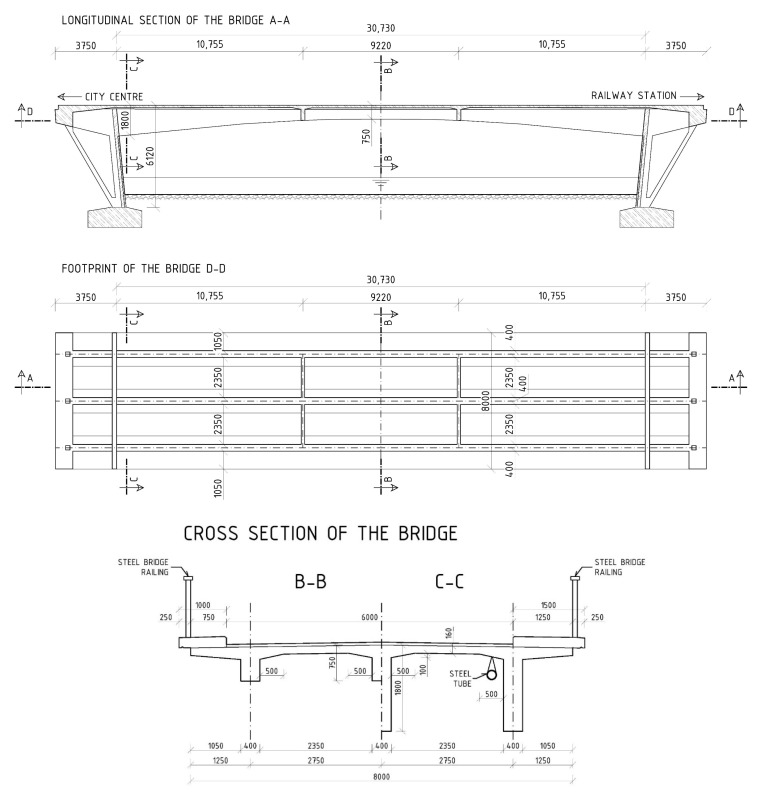
Schemes of the bridge (dimensions are in [mm]).

During the summer of 2021, a diagnostic survey was performed on the post-tensioned bridge in the center of Banska Bystrica (central Slovakia), see Figure 3. It was a monolithic supporting structure consisting of three separate beams, struts, and additional prestressed ties. The schemes of the investigated bridge can be seen in Figure 4. Based on available documents, it is known that the bridge in question was built in 1959, so it was 62 years old at the time of the test. A diagnostic survey was performed in order to verify the state of the bridge, which originally carried the road traffic over the river Hron. In the future, the idea of the preservation of the bridge is considered only for pedestrian traffic.

The structure of the analyzed bridge is quite uncommon for the period of construction. The bridge acts as a frame structure with ties and struts on both sides of the superstructure. The superstructure is formed by a variable cross-section of the beam with a height from 1800 to 750 mm and a width of 400 mm. The thickness of the concrete slab connecting three beams is variable from 160 to 260 mm, while the total width of a bridge is 8000 mm. Moreover, there are two sidewalks on the bridge. For a better interaction and distribution of the load, the beams are stiffened by two transversal girders at a distance of 10,755 mm from the struts of the bridge. Sloping struts are approximately 6000 mm high with a thickness of 300 mm. Ties are also sloping and connect the ends of the beams and the footings. Their length is 4800 mm. The riverbed under the bridge is paved with rocks. The overall length of the bridge is 38,230 mm and the clear span is roughly 30,000 mm. More information about the dimensions of the bridge is presented in Figure 4. 

The concrete’s compressive strength (which is not strictly required for SCM evaluation) was roughly determined using the non-destructive Schmidt hammer rebound method, according to Equation (1). Moreover, obtained results were used for the estimation of the modulus of elasticity of the concrete using Equation (2). The results are presented in Table 1 and Table 2. The used technique is suitable for in situ basic evaluations. The assessment of the concrete’s compressive strength, obtained according to the reading from the measurement using the Schmidt hammer method, was performed during the diagnostic survey. Specifically, it was performed in three different places on the beam. As a result, the concrete strength class was determined according to the in situ evaluation, which was based on today’s applicable standards [22,23,24]. The evaluation indicated the beam’s concrete strength class of C40/50.

β_n_ = 1.98; s_r_ = 1.78

R_bg_ = R_be,i,avg_ − β_n_ × s_r_ = 55.34 MPa → C40/50 (1)

E_cm_ = 22 × (f_cm_/10) ^0.3^ = 22 × (48/10) ^0.3^ = 35.22 GPa (2)

**Figure 5:**
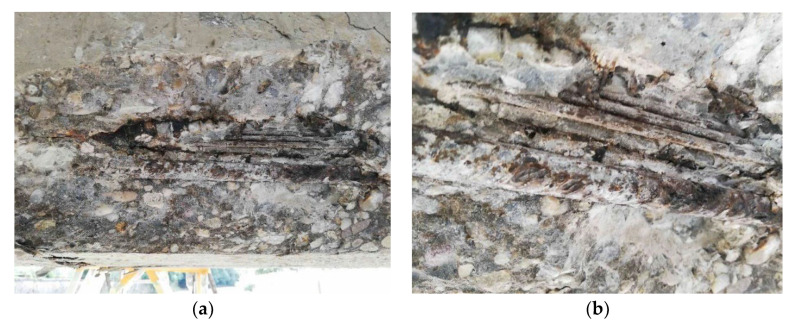
Prestressing wires after exposure in the midspan area—view (**a**); detail (**b**).

**Figure 6:**
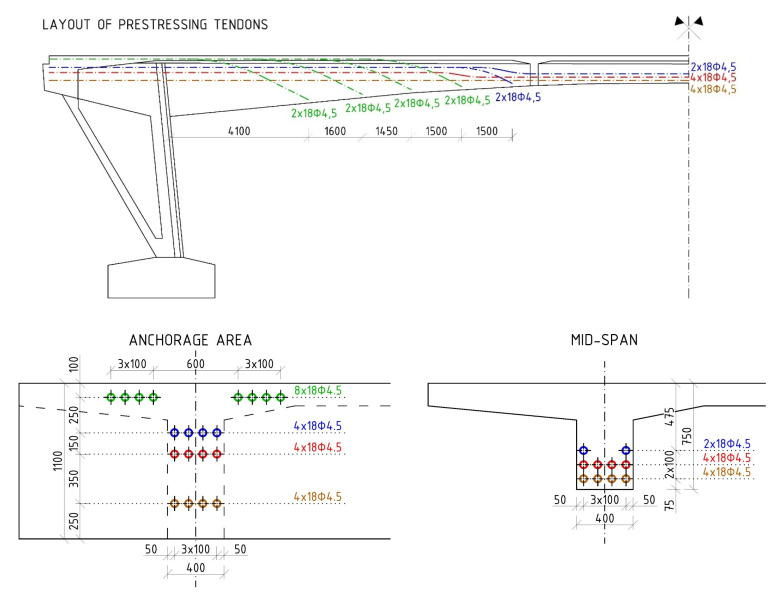
The layout of prestressing tendons (dimensions are in [mm]).

The material parameters of the prestressing steel were determined according to available documents (so-called preliminary static assessments) and the era of construction—the tensile strength of the prestressing wires, f_p_, of 1650 MPa and the yield strength, f_y_, of 1080 MPa. The modulus of elasticity of the patented prestressing wires (E_p_) was 190 GPa. As a part of the diagnostic survey, prestressing tendons were destructively exposed in the midspan area. Consequently, the corrosion of prestressing wires was assessed as insignificant, see Figure 5. Signs of cement grout have been seen in the central part of the beam and also in the anchorage area. After exposure of anchorage area, we have found out that the tendons consisted of eighteen patented wires with a diameter of 4.5 mm. The layout of the prestressing tendons is presented in Figure 6. Only continuous prestressing tendons were anchored in the ends of the beams, while non-continuous tendons’ anchorages were located in the lower edge of the beams in the distance of approximately 1.50 m.

**Figure 7:**
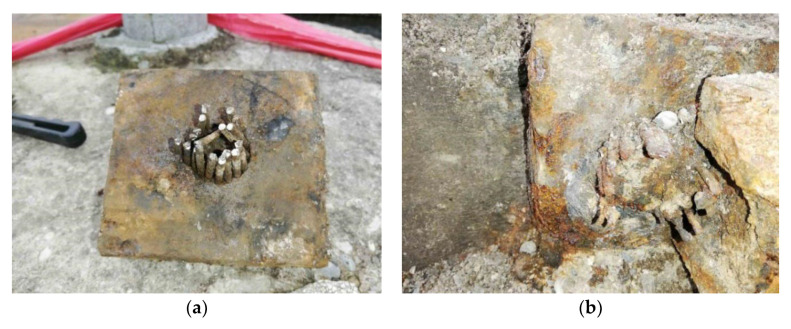
Anchorages—removed anchorage (**a**); detail (**b**).

**Figure 8:**
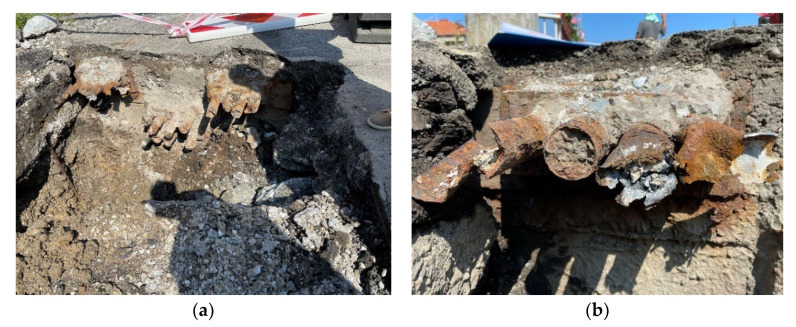
Anchorage area at the end of the beam—view (**a**); detail (**b**).

Anchoring of the prestressing tendons was provided by the so-called Horel anchoring wedge system, based on the Freyssinet principle. This system was commonly used in former Czechoslovakia at the time of the construction of the assessed bridge [1]. During the diagnostic survey, the anchorage areas on both sides of the bridge were exposed. The anchorages on the side of the bridge which was closer to the city center were in worse conditions than the anchorages by the railway station. The anchorages can be seen in Figure 7 and Figure 8.

## 3. Experimental Program

The experimental program consisted of three individual methods, for which the outputs were later evaluated and compared.

### 3.1. Saw-Cut Method

**Figure 9:**
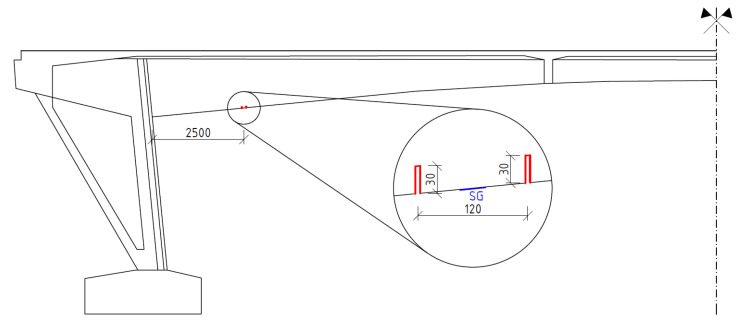
Position of performed saw-cuts and their parameters (dimensions are in [mm]).

**Figure 10:**
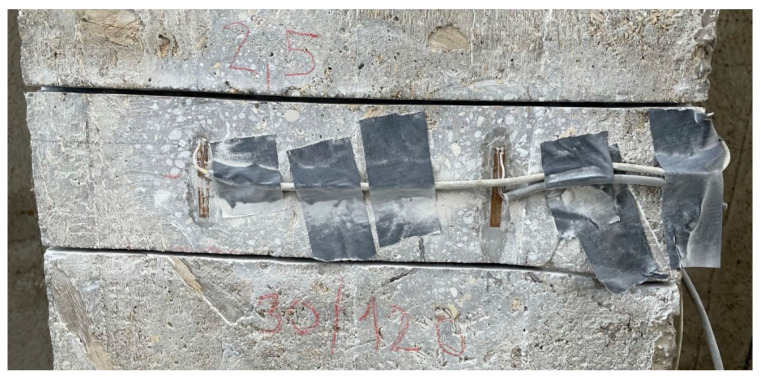
View on performed saw-cuts and installed strain gauges.

One pair of saw-cuts with an axial distance of 120 mm and depth of 30 mm was applied on the bottom edge of the central beam at the distance of 2.50 m from the strut of the bridge, see Figure 9. When using saw-cut method, our intention should be to avoid cutting the beam’s reinforcement. Thus, we decided to use 30 mm-deep saw-cuts. During the measurement, two strain gauges of type HBM LY41-50/120 were used to record the change in strain. These strain gauges’ measuring grid length was 0.05 m. Strain gauges were positioned in the center between the saw-cuts. Transversely, they were installed symmetrically on the bottom edge of the post-tensioned member. Saw-cuts and used strain gauges are shown in Figure 10. Unfortunately, during the experimental measurement, one of the strain gauges (SG2) was damaged. Consequently, only normal stress (strain) change reading from strain gauge SG1 is considered in the evaluation of saw-cut method. The experimental program of saw-cut method was finished after the recorded values of change in normal stress were stabilized. The maximum value of normal stress release Δσ_c_ (considering modulus of elasticity of concrete of 35.22 GPa) measured by the strain gauge SG1 was 4.10 MPa.

### 3.2. Structural Response Method

**Figure 11:**
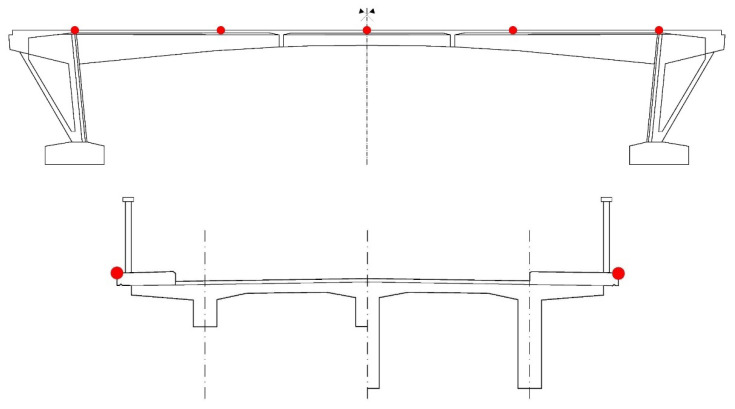
Position of monitoring points for Geodetic Surveying.

**Figure 12:**
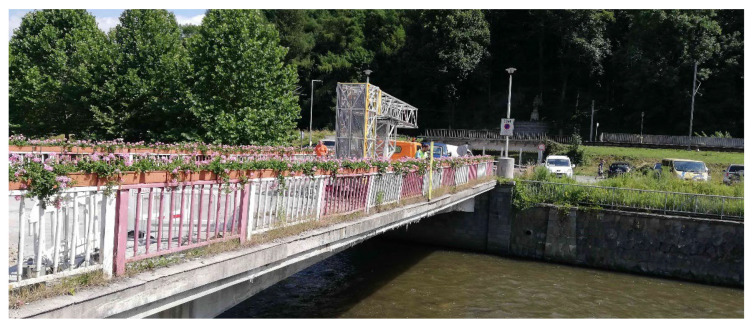
Geodetic Surveying—Measurement.

As a part of the experimental measurements, analysis using structural response method was performed too. Response of structure on dead load was represented by deflection measured using Geodetic Surveying in five different points on both sides of the cross-section of the bridge (left and right kerbs/parapets). Subsequently, the deflection of the bridge was determined as the average value. Following positions of monitoring points were chosen—points over struts, quarters of the effective span, and midspan. Monitoring points are presented in Figure 11. The measurement of the deflection of the bridge can be seen in Figure 12. The application of structural response method on the PC bridge was based on the measurement of deflection in five different points along the span of the bridge and subsequent comparison of obtained results to numerical analysis.

### 3.3. Barkhausen Noise Technique

This technique was used only as a validation method on one prestressing wire in the midspan area and was performed after the detection of prestressing steel during the diagnostic survey. MBN was measured by RollScan 350 in MicroScan 600 software (magnetizing voltage 5 V and frequency 125 Hz of sine profile (the corresponding magnetizing field altered ± 4.64 kA·m^−1^)). The sampling frequency of acquired MBN emission was 6.40 MHz. Magnetic field was altered over time along the wire length. Further details about the sensor shape and its positioning can be found in [19]. MBN signal was acquired in the frequency range from 10 to 1000 kHz by the use of serial sensor S1-18-12-01. MBN refers to the rms value of the signal. In order to obtain a calibration curve (evolution in which MBN is plotted as a function of tensile stress), one wire was cut off stressed and then stressed by the use of device Instron 5985 [25].

## 4. Numerical Analysis

Numerical analysis (NA), which was necessary for the evaluation of the experimental measurements, was performed in ATENA 2D Software (version ATENA 5.7.0n, Červenka Consulting, Prague, Czech Republic) with the support of Time Dependent Analysis (TDA) executed in the software SCIA Engineer (version 20.0.4012.32, Nemetschek Group, Munich, Germany). These assumptions of material properties were assigned to macro-elements in numerical software: Concrete (SBeta Material) − f_c,cube_ = 50.0 MPa; f_c,cyl_ = 42.5 MPa; f_t_ = 3.257 MPa; E_c_ = 35,220 MPa; ν = 0.20; Prestressing Steel (Reinforcement–Bilinear) − f_y_ = 1,080 MPa; E_p_ = 190,000 MPa; saw-cuts (Plane Stress Elastic Isotropic) − E_SC_ = 1.0 kPa; ν = 0.30.

### 4.1. Time-Dependent Analysis in SCIA Engineer

**Figure 13:**
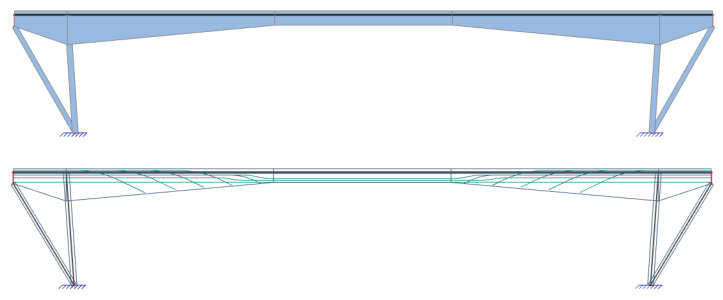
FE model used for TDA analysis.

Analysis was performed in a 2D environment which enables Time-Dependent Analysis considering the rheology of concrete and technology of construction [26,27]. The Finite Element (FE) model is shown in Figure 13. Therefore, only one beam with a corresponding load was modeled. This analysis was essential for the determination of the contribution of rheology (creep and shrinkage) to deflection after 62 years of service of the assessed bridge. Moreover, the FE model provided data about the expected stress in each individual tendon at the time of testing.

**Figure 14:**
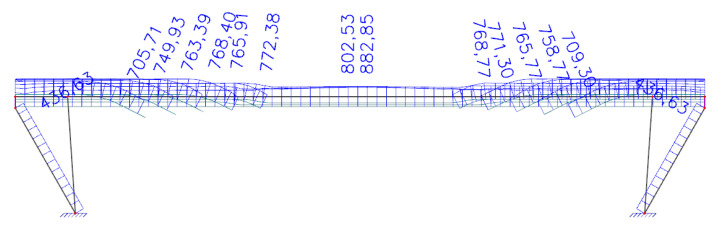
Stress [MPa] in tendons after 62 years of service.

**Figure 15:**
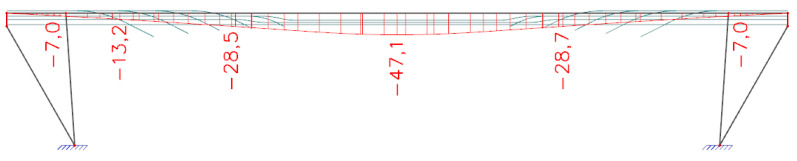
Deflection [mm] resulting from the rheology of concrete after 62 years of service.

Initial stress during the tensioning of prestressing tendons was assigned with a value of 1000 MPa in compliance with standards used at the time of the design of the bridge and according to available documents provided by the administrator. *Analysis of a bridge which is in service for a long time is always a challenge because the input data are usually not available, or we have only brief information.* Consequently, *in the presented analysis, our assumptions were based on the original static analysis which we obtained from the administrator.* The software enables one to estimate the prestress losses according to today’s applicable Eurocode 2 [24]. In TDA, the end of the concrete curing after 72 h was considered. The relative humidity of the environment was assigned with a value of 70% and the anchorage draw-in was considered with a value of 6 mm, the coefficient of friction between the tendon and its duct had a value of 0.30, and an unintended regular displacement for the internal tendons had a value of 0.005. All tendons were assigned one active anchorage. The additional loads resulting from the self-weight of pavement, sidewalks, and railings were taken into account by a load with a value of 8.44 kN·m^−1^. This load was assigned as uniformly distributed. Additionally, the self-weight of transversal beams connecting longitudinal beams were considered by forces with the value of 6.50 kN. The supports were assigned as fixed. According to records, the bridge in question was built using a scaffolding system as the paved riverbed of the Hron river enabled this building technology. Thus, performed TDA considered this fact. The stress in individual tendons after 62 years of service, taking into account all the aforementioned parameters, is presented in Figure 14. According to the TDA, the stress in the tendons, calculated as a weighted average in the midspan, should be 829 MPa (analysis using MBN), and in the cross-section at a distance of 2.50 m from the strut (application of saw-cuts), it should have a value of 776 MPa. Deflection resulting only from the rheology of concrete can be seen in Figure 15. Consequently, TDA suggests that the rheology of concrete contributed to the overall midspan deflection with a value of −47.1 mm.

### 4.2. Nonlinear Numerical Analysis in ATENA 2D Software

**Figure 16:**
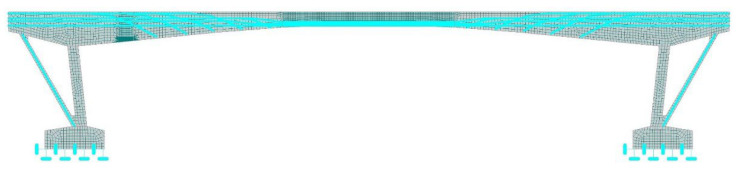
FE model in ATENA 2D Software.

In the analysis, one post-tensioned monolithic beam was modeled as a 2D finite element. The numerical model is displayed in Figure 16. Similarly, as in the previous TDA analysis, the loads resulting from the self-weight of the pavement, sidewalks, and railings were assigned to a value of 8.44 kN·m^−1^. All these loads were taken into account as uniformly distributed loads. Furthermore, the self-weight of the transversal beams connecting the longitudinal beams was represented by forces with the value of 6.50 kN. The line supports were modeled on the bottom edges of the footings carrying the whole frame structure of the analyzed bridge. According to the diagnostic survey, the cement grout was discovered in the tendons. Therefore, the analysis considered these findings. The prestressing force in individual tendons was assigned according to the results of the already mentioned TDA. Thus, the long-term effect of the rheology and other factors affecting prestress losses on the normal stress state in PC members was taken into consideration.

In the middle area of the beam, the FE mesh was smoothed into quadrilateral CCQ10SBeta 50 mm large elements. The reason was that, in this part of the beam, the diagnostic survey indicated crack propagation resulting even from the dead load. The same mesh size was attributed to the area which was close to the saw-cuts. Moreover, the part of the beam directly close to the performed saw-cuts was smoothed into 8-mm-large elements. The rest of the modeled beam was assigned identical 150 mm quadrilateral CCQ10SBeta elements. The sawing was modeled by means of applying the so-called “construction stages”. Firstly, all macro-elements were assigned material properties of the surrounding concrete. In the second stage, the modulus of elasticity of the macro-elements that characterized the saw-cuts was significantly decreased.

**Figure 17:**
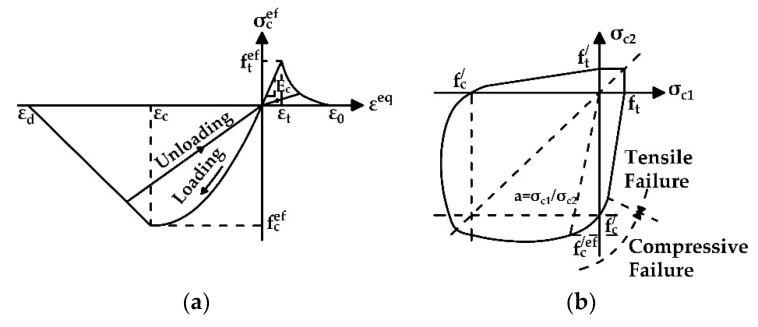
(**a**) Stress–strain curve for concrete; (**b**) biaxial failure function for concrete.

**Figure 18:**
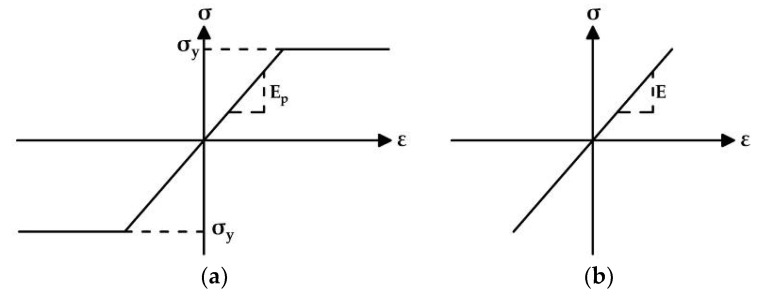
(**a**) Stress–strain curve for prestressing steel; (**b**) stress–strain curve for saw-cuts.

The SBeta constitutive model of concrete includes twenty material parameters. Simulations consider known parameters of materials. However, performed material testing did not provide all the required values for the numerical analysis. Therefore, the remaining parameters were assigned according to ATENA 2D Software guidelines for FE analysis. The formulation of constitutive relations is considered in the plane stress state. The stress–strain curves of individual modeled materials are shown in Figure 17 and Figure 18. Further information about the SBeta constitutive model can be found in [28,29,30].

#### 4.2.1. Saw-Cut Method

**Figure 19:**
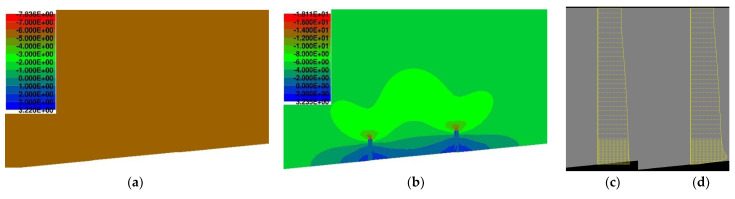
Normal stress [MPa] distribution—before sawing (**a**,**c**); after sawing (**b**,**d**).

Normal stress values were determined in nine monitoring points over a total length of 50 mm, which expresses the length of the measuring grid of the used strain gauges during the experimental campaign. Subsequently, the normal stress relief (Δσ_c_) was calculated as the average value from all individual monitoring points. The initial normal stress values (σ_c,0_), normal stress values after the application of saw-cuts with a depth of 30 mm (Δσ_c,30_), and values of normal stress relief (Δσ_c_) are listed in Table 3. According to numerical analysis, the total value of normal stress relief was −4.49 MPa. Results of the nonlinear numerical analysis suggest that the performed saw-cuts (axial distance of 120 mm, depth of 30 mm) initiated circa 80% stress relief. This agrees with the findings of the parametric study presented in [31] or in [32]. The distribution of normal stress in the area adjacent to the performed saw-cuts is shown in Figure 19.

#### 4.2.2. Structural Response Method

**Figure 20:**
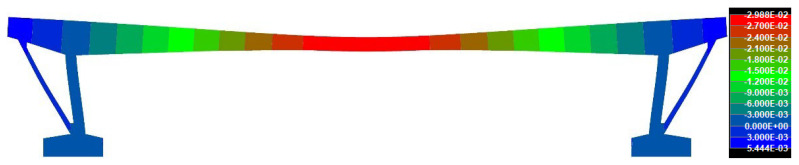
Deflection [m] resulting from the dead load (excluding rheology of concrete).

Numerical analysis (NA) of the SRM was based on the determination of the deflection which resulted from the dead load, excluding the rheology of the concrete. The effect of rheology on the deflection of the bridge was calculated in the aforementioned TDA in the software SCIA Engineer. Accordingly, NA in ATENA 2D Software suggests that the dead load, excluding the rheology of the concrete, caused a deflection of −29.9 mm. A deformed structure can be seen in Figure 20.

## 5. Discussion

### 5.1. Saw-Cut Method

**Figure 21:**
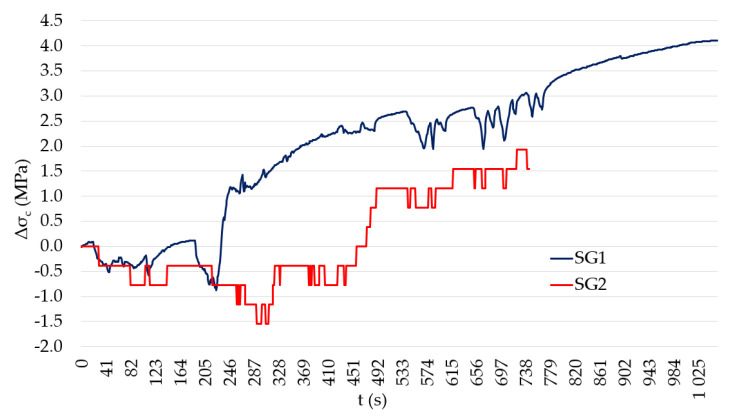
Measured normal stress relief after application of saw-cuts (SG2 was damaged).

The evaluation of the state of prestressing consisted of a comparison of experimentally measured values and numerical analysis. Normal stress readings after the application of a pair of 30 mm-deep saw-cuts, which were performed at a distance of 120 mm, are displayed in Figure 21. It is evident that such an intervention into this investigated PC member releases normal stress of 4.10 MPa. This value shows good agreement (91.3%) with the numerical analysis which provided the value of 4.49 MPa. Insignificant discrepancies can be attributed, for example, to errors of measuring instruments, the inaccurate determination of material properties, and so on. Moreover, numerical analysis (NA) suggests that the performed saw-cuts caused approximately 80% normal stress relief. Therefore, it can be assumed that initial normal stress in the investigated cross-section was circa −5.13 MPa (NA: −5.60 MPa).

**Figure 22:**
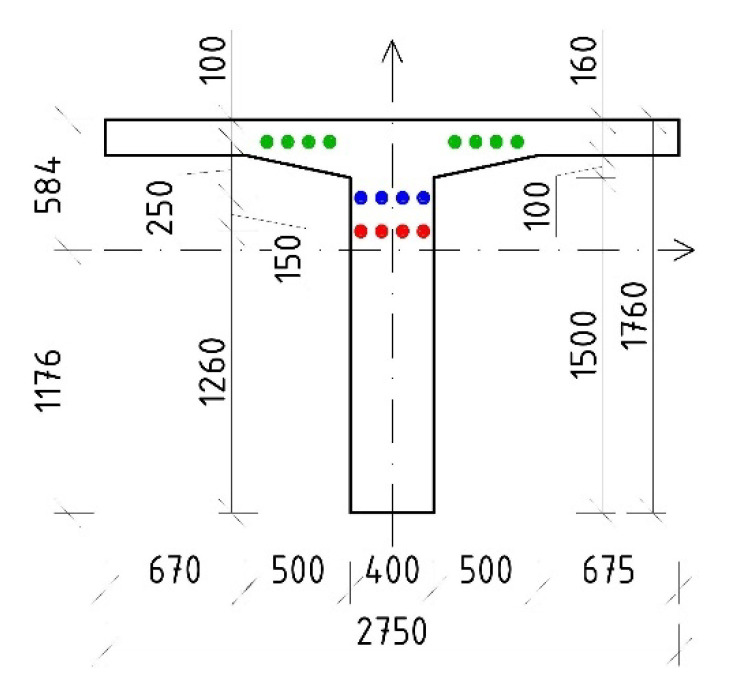
Ideal cross-section of the investigated beam (2.50 m from the strut), dimensions are in [mm].

Finally, the evaluation based on the results of the saw-cut method was completed using the assumption of the linear distribution of normal stress. Hence, the residual prestressing force after 62 years of service (P_m62,residual_) in the studied bridge was calculated using Equation (3), where σ_c,0_ = −5.13 MPa represents the initial normal stress in the concrete member (cross-section at a distance of 2.50 m from the strut), M_G_ = −543 kNm is the bending moment due to the dead load (including the rheology of concrete), and e_pi_ is the eccentricity of the prestressing reinforcement from the neutral axis of an ideal cross-section. Characteristics of the ideal cross-section are represented by I_yi_ = 3.432 × 10^11^ mm^4^ (second moment of inertia), A_i_ = 1,098,494 mm^2^ (cross-sectional area), and z_bi_ = 1176 mm (position of neutral axis from the bottom edge of the ideal cross-section). Finally, the value of the residual prestressing force, according to the outputs from the saw-cut method, was −3480 kN. This implies that the average stress of the tendons in the cross-section at a distance of 2.50 m from the strut is approximately 760 MPa (overall area of prestressing steel of A_p_ = 4580.4 mm^2^). In comparison, TDA provided an average value of the stress (weighted average) in the tendons in the evaluated cross-section after 62 years of service of 776 MPa (agreement by 97.9%). Ideal cross-sectional characteristics used in the evaluation are listed in Table 4, whereas the dimensions of the ideal cross-section can be seen in Figure 22.

P_m62,residual_ = − [ σ_c,0_ − (M_G_/I_yi_) × z_bi_]/[(1/A_i_) + (e_pi_/I_yi_) × z_bi_] = −3480 kN (3)

### 5.2. Structural Response Method

**Figure 23:**
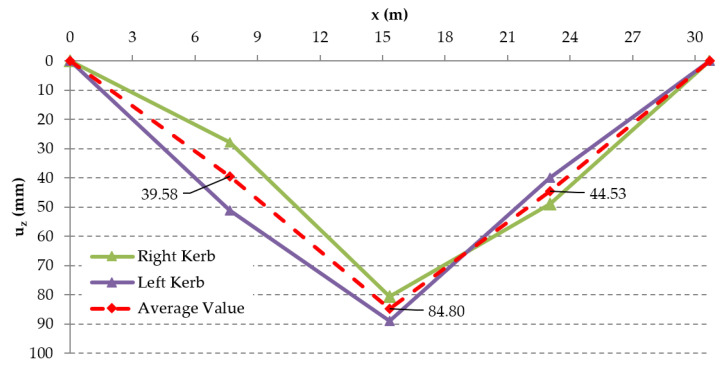
Measured deflections of the bridge using Geodetic Surveying.

The structural response method was used to validate the conclusions of the saw-cut method. The deflections measured in the overall five points (over struts, quarters of the effective span, and midspan) were evaluated as average values because the Geodetic Leveling was performed on both sides (kerbs) of the bridge. The results suggest that the deflection in the quarters of the span were −39.58 mm (railway station) and −44.53 mm (city center). The average deflection in the midspan was −84.80 mm. The determination of deflection from the numerical analysis was divided into two parts—deflection generated by the long-term effect of the rheology of concrete (TDA in SCIA Engineer) and deflection resulting from the dead load (nonlinear analysis in ATENA 2D Software). TDA analysis provided the value of −29.9 mm, while nonlinear analysis suggest deflection of −47.1 mm. Therefore, the total deflection from NA was −77.0 mm (agreement with experimental measurement is by 90.8%). That means that the state of prestressing of the assessed bridge after 62 years of service is very similar to the theoretically determined residual prestressing force using an approach according to today’s applicable Eurocodes [24]. Additionally, in this case, disagreements can be credited to, for example, errors of measuring instruments, insecurities in models, etc. The measured deflection curve is shown in Figure 23.

### 5.3. Barkhausen Noise Technique

**Figure 24:**
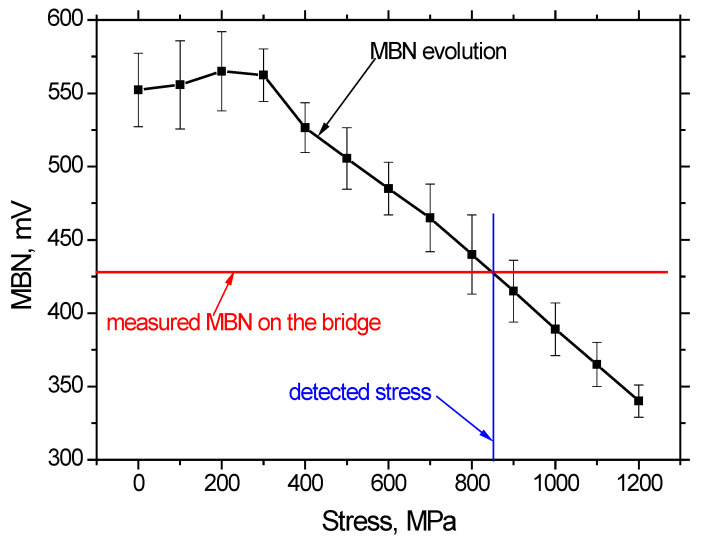
Evolution of MBN versus tensile stress state together with assessment of the true stress of prestressed wire.

This approach was performed in the midspan area after the exposure of prestressing wires which were part of the diagnostic survey. Figure 24 depicts the evolution of MBN along with the increasing amplitude of tensile stresses. This figure clearly confirms that the energy of magnetocrystalline anisotropy is fully consumed by magnetostriction energy at higher stresses. For this reason, MBN drops down when the amplitude of tensile stresses grows. Furthermore, this figure also depicts that the intersection between this evolution and the line representing the MBN measured directly in the prestressed state on the bridge is about 850 MPa, which confirms the calculations indicated above. 

## 6. Conclusions

The presented experimental state of prestressing analysis is supported by numerical simulations aimed to verify the residual prestressing force using three different techniques—the saw-cut method (SCM), the structural response method (SRM), and the Barkhausen noise technique (MBN). *The bridge in question has not been monitored since its construction. Therefore, we evaluated the deflection measured at the time of the diagnostic survey. For the evaluation, we assumed that the obtained value considered the deflection resulting from the dead load and time-dependent phenomena after 62 years. If the investigated bridge was continuously monitored throughout its service life, the analysis would be more accurate. Unfortunately, such local bridges are not usually continuously monitored. In practice, it is almost impossible to determine the precise residual level of prestressing without reliable input data.* Based on the acquired findings, the following conclusions can be summarized:

The saw-cut method (SCM) enabled us to determine the initial normal stress in the cross-section at a distance of 2.50 m from the strut. Subsequently, the calculation suggested that the value of the residual prestressing force is approximately −3480 kN. As a result, the stress in the tendons in the monitored cross-section was 760 MPa. Numerical analysis performed in compliance with today’s applicable standards provided an average stress in tendons of 776 MPa. Therefore, the agreement of the experimental measurement with the numerical analysis is adequate.

The experimental measurement using the structural response method (SRM), based on the monitoring of the long-term effect of a dead load including the rheology of concrete, provided the value of deflection in the midspan of −84.80 mm. Subsequently, Time-Dependent Analysis and nonlinear analysis indicated that the deflection, after 62 years of service, according to Eurocodes, should be −77.0 mm, so the consistency with the experiment is satisfactory.

Validation of the state of prestressing practicing the Barkhausen noise technique (MBN) provided a stress value, in the investigated prestressing wire, of approximately 850 MPa. According to numerical analysis, the average stress in the tendons in the midspan area was 829 MPa. Hence, also in this case, the experimental measurement confirmed the assumptions of the numerical analysis.

One of the outcomes from the application of the saw-cut method (SCM) is that a relatively small intervention in the assessed structure can provide valuable data about the normal stress state in the investigated structure. As the analysis proved, saw-cuts with an axial distance of 120 mm and a depth of 30 mm caused almost 100% of the normal stress release. In the case of an investigated post-tensioned member, it was an 80% change in normal stress. Such an intervention does not influence the integrity of the structure because the saw-cut can be easily and properly fixed.

Among the benefits of the introduced approaches is that they can be accomplished, also, only under a permanent load. Due to this point, if the assessed prestressed concrete structure is unloaded, the state of the prestressing analysis is not problematic. The presented experimental measurements can be applied rapidly on site. Moreover, sophisticated tools are not needed.

As a conclusion for the administrator, the state of prestressing of the investigated bridge is satisfactory considering the length of its service life. Therefore, the idea of the usage of this bridge for pedestrian traffic in the future is realistic because the analysis confirmed the decent state of the structure, including the prestressing tendons. However, certain minor interventions should be planned. The administrator should focus on the retrofitting of the structure and anchorage area. These actions would contribute to the higher durability of the bridge in question and help to extend its service life. From the theoretical and also practical point of view, the presented analysis suggests that it is possible, by means of a combination of in situ measurements and subsequent numerical analysis, to evaluate the overall state of the prestressing of existing prestressed concrete structures. Since most of these methods are still under development, it is necessary to apply several techniques and compare their outputs. This is the only way to check the obtained results.

Our findings could help in the assessment of similar prestressed concrete bridges (or structures). The comparison of results obtained using several different approaches is always the best option in the evaluation of the residual prestressed concrete structure, as the prestressing force value is the crucial data for the subsequent determination of the load-carrying capacity.

## Figures and Tables

**Table 1:** Schmidt hammer rebounds.

Testing Place	Schmidt Hammer Rebound a (-)					a_avg_. (-)	R_ci_ (MPa)					R_ci,avg_ (MPa)	α_t_ (-)	α_w_ (-)	R_be,i_ (MPa)
S1	56	54	50	50	58	53.6	69	67	59	59	75	65.8	0.9	1.0	59.2
S2	56	54	56	52	54	54.4	69	67	69	63	67	67.0	0.9	1.0	60.3
S3	52	51	52	52	54	52.2	63	61	63	63	67	63.4	0.9	1.0	57.1

R_be,i,avg_ = 58.86 MPa.

**Table 2:** Evaluation of Schmidt hammer rebounds.

i	R_be,i_ (MPa)	R_be,i,avg_ (MPa)	R_be,i_—R_be,i,avg_ (MPa)	(R_be,i_—R_be,i,avg_)^2^ (MPa^2^)
1	59.22	58.86	0.36	0.13
2	60.30	58.86	1.44	2.07
3	57.06	58.86	−1.80	3.24

**Table 3:** Normal stress values from numerical analysis performed in ATENA 2D Software.

Point	σ_c,0_ (MPa)	σ_c,30_ (MPa)	Δσ_c_ (MPa)	Δσ_c_ (%)
1	−5.58	−0.71	−4.87	87
2	−5.58	−1.09	−4.50	81
3	−5.58	−1.09	−4.50	81
4	−5.59	−1.30	−4.29	77
5	−5.59	−1.30	−4.29	77
6	−5.60	−1.32	−4.28	76
7	−5.61	−1.17	−4.44	79
8	−5.61	−1.17	−4.44	79
9	−5.63	−0.84	−4.79	85
**Avg.**	**−5.60**	**−1.11**	**−4.49**	**80**

**Table 4:** Ideal cross-sectional characteristics (2.50 m from the strut).

A_i_ (mm^2^)	I_yi_ (mm^4^)	z_bi_ (mm)	z_ti_ (mm)	e_pi_ (mm)
1,098,494	3.432 × 10^11^	1176	584	321

## Data Availability

Not applicable.

## Nomenclature

SC	saw-cut,
PC	prestressed concrete,
FE	finite element,
SG	strain gauge,
MBN	magnetic Barkhausen noise, Barkhausen noise technique,
SRA	Slovak Road Administration,
SCM	saw-cut method,
SRM	structural response method,
E_p_	modulus of elasticity of prestressing steel (MPa),
f_p_	tensile strength of prestressing steel (MPa),
f_y_	yield stress of prestressing steel (MPa),
NA	numerical analysis,
TDA	time-dependent analysis,
f_c,cyl_	cylindrical compressive strength of concrete (MPa),
f_c,cube_	cubic compressive strength of concrete (MPa),
f_t_	tensile strength of concrete (MPa),
ν	Poisson’s ratio (-),
σ_c,0_	initial stress in concrete (MPa),
σ_c,30_	stress in concrete after application of saw-cuts (MPa),
Δσ_c_	change in stress (MPa),
ΔP_m62,residual_	residual prestressing force (kN),
A_i_	ideal cross-sectional area (m^2^),
A_p_	cross-sectional area of prestressing steel (m^2^),
e_pi_	ideal eccentricity of prestressing force from the neutral axis (m),
I_yi_	ideal second moment of inertia (m^4^),
z_i_	position of the neutral axis of an ideal cross-section from the edge (m),
M_G_	moment due to dead load (kNm).
